# Association Between Changes in Post-hospital Cardiac Symptoms and Changes in Acute Coronary Syndrome-Induced Symptoms of Post-traumatic Stress

**DOI:** 10.3389/fcvm.2022.852710

**Published:** 2022-04-14

**Authors:** Roland von Känel, Rebecca E. Meister-Langraf, Claudia Zuccarella-Hackl, Hansjörg Znoj, Aju P. Pazhenkottil, Jean-Paul Schmid, Jürgen Barth, Ulrich Schnyder, Mary Princip

**Affiliations:** ^1^Department of Consultation-Liaison Psychiatry and Psychosomatic Medicine, University Hospital Zurich, University of Zurich, Zurich, Switzerland; ^2^Clienia Schlössli AG, Zurich, Switzerland; ^3^Department of Health Psychology and Behavioral Medicine, University of Bern, Bern, Switzerland; ^4^Department of Cardiology, University Hospital Zurich, University of Zurich, Zurich, Switzerland; ^5^Cardiac Imaging, Department of Nuclear Medicine, University Hospital Zurich, University of Zurich, Zurich, Switzerland; ^6^Department of Internal Medicine and Cardiology, Clinic Gais AG, Gais, Switzerland; ^7^Institute for Complementary and Integrative Medicine, University Hospital Zurich, University of Zurich, Zurich, Switzerland; ^8^Medical Faculty, University of Zurich, Zurich, Switzerland

**Keywords:** cardiovascular disease, enduring somatic threat, interoception, psychological stress, somatic symptoms, trauma

## Abstract

**Background:**

After acute coronary syndrome (ACS), one in eight patients develops clinically significant symptoms of Post-traumatic stress disorder (PTSD). We hypothesized that changes in cardiac symptoms from 3 to 12 months after ACS are associated with changes in ACS-induced PTSD symptoms.

**Methods:**

At 3 (*n* = 154) and/or 12 months (*n* = 106) post-ACS, patients (*n* = 156, mean age 59 years, 85% men) completed a clinical interview assessing chest tightness/pain (at rest and/or during exertion), heartbeat symptoms (heart palpitations, racing of heart, heart stumbling or skipping a beat) and PTSD symptoms during the prior 4 weeks. Random mixed regression models examined the association between the onset (or remission) from 3 to 12 months in cardiac symptoms with changes in PTSD symptoms, adjusting for a range of potential predictors of ACS-induced PTSD symptoms.

**Results:**

The onset of chest tightness/pain [estimate = 0.588, 95% confidence interval: 0.275, 0.090; *p* < 0.001] and of heartbeat symptoms [0.548 (0.165, 0.931); *p* = 0.005] from 3 to 12 months was independently associated with an increase in total PTSD symptoms. There were also independent associations between the onset of chest tightness/pain and heartbeat symptoms with an increase in PTSD symptom clusters. Specifically, the onset of chest tightness/pain showed associations with an increase in re-experiencing [0.450 (0.167, 0.733); *p* = 0.027] and avoidance/numbing [0.287 (0.001, 0.574); *p* = 0.049]. The onset of heartbeat symptoms showed associations with an increase in re-experiencing [0.392 (0.045, 0.739); *p* = 0.002], avoidance/numbing [0.513 (0.161, 0.864); *p* = 0.004] and hyperarousal [0.355 (0.051, 0.659); *p* = 0.022]. An increase in the total number of cardiac symptoms (score range 0–6) was also associated with an increase in total PTSD symptoms [0.343 (0.202, 0.484); *p* < 0.001]. Psychotherapy in the post-hospital period moderated the association between the change in heartbeat symptoms and the change in total PTSD symptoms [−0.813 (−1.553, −0.074); *p* = 0.031 for interaction]; the association between the onset of heart beat symptoms and an increase in total PTSD symptoms was weaker in patients who attended psychotherapy [0.437 (−0.178, 1.052); *p* = 0.16] than in those who did not [0.825 (0.341, 1.309); *p* < 0.001].

**Conclusion:**

Changes in cardiac symptoms between 3 and 12 months after hospitalization are associated with changes in ACS-induced PTSD symptoms. ClinicalTrials.gov #NCT01781247.

## Introduction

Post-traumatic stress disorder (PTSD) is a debilitating mental disorder, which can be induced by serious medical conditions that are accompanied by threatened death and physical integrity (1). In the wake of acute coronary syndrome (ACS), about 12% of patients develop clinically significant PTSD symptoms (2). Typical PTSD symptoms include re-experiencing aspects of ACS in thoughts or dreams along with physical reactions, avoiding reminders of ACS, such as places where the event occurred, and hyperarousal, including startle reactions (3). Even with subclinical expressions of ACS-induced PTSD symptoms, there is an increased risk of recurrent cardiovascular events and mortality (2), which may be higher for the re-experiencing symptom cluster than for the other symptom clusters (4). The increased cardiovascular risk may partially be explained by patients avoiding medication and physical activity to reduce traumatic reminders of the cardiac event (5, 6). Unfortunately, there is currently insufficient evidence from randomized controlled trials that early interventions to prevent the development of PTSD after medical events are effective (7). Specifically, in the Myocardial Infarction-Stress Prevention Intervention (MI-SPRINT) randomized controlled trial, we found that one single session of trauma-focused counseling applied within 48 h of hospital admission was not more effective than stress-focused counseling in preventing the development of clinician rated ACS-induced PTSD symptoms at 3 and 12 months (8, 9). Therefore, to improve patients' quality of life and prognosis with targeted early interventions, it is critical to understand the mechanisms contributing to ACS-related PTSD symptoms (7).

In patients with ACS, cardiac symptoms are one potential proximal target to improve medical outcomes and quality of life in general and to reduce PTSD symptom development in particular. Somatic symptoms that occur and persist after traumatic experiences with physical injury or as physical stress reactions are increasingly recognized as a potentially important clinical entity contributing to PTSD symptoms. For instance, early somatic pain and other symptoms, including palpitations, predicted PTSD symptoms in survivors of a terrorist shooting (10), and somatic symptoms, including shortness of breath, predicted overall PTSD symptoms 6 months after an earthquake (11). In patients with ACS, recurrence of cardiac symptoms, chiefly chest pain, in the post-hospital period predicted PTSD symptoms at 12 months, as somatic symptoms could act as vivid reminders of the event (12). As internal somatic cues, chest tightness/pain and the heart beating irregularly may represent ongoing somatic threat contributing to PTSD symptoms (13, 14). This may particularly be the case if patients have high peritraumatic threat perceptions in the form of fear of dying and feelings of helplessness during ACS (14). Elevated levels of threat perceptions are reported by 20–25% of patients evaluated for ACS (15, 16) and are predictive of PTSD symptoms (17, 18) and recurrent cardiovascular events (19). The enduring somatic threat model of PTSD integrates these and other research findings and posits that the underlying fear of mortality perpetuates PTSD symptoms due to both discrete/external and ongoing/somatic events after a life-threatening medical event (13). Additional support for this model is provided by a recent prospective study in patients with an acute cerebrovascular event in which stroke symptom severity (i.e., internal somatic reminders of the event) measured before hospital discharge was associated with PTSD symptoms 1 month later (20).

Based on this literature, we aimed to elucidate the association between changes in cardiac symptoms in the post-hospital period with changes in ACS-induced PTSD symptoms using data collected in the MI-SPRINT study mentioned above (8, 9, 21). We specifically hypothesized that the onset from 3 to 12 months in chest tightness/pain and heartbeat symptoms would be associated with an increase in total ACS-induced PTSD symptoms, independent of a number of known predictors of ACS-induced PTSD symptoms (22). We also explored these longitudinal associations for the three PTSD symptom clusters re-experiencing, avoidance/numbing, and hyperarousal, because they may be differently related to cardiovascular risk (4). Moreover, we examined whether greater peritraumatic threat perceptions, use of cardiac rehabilitation or psychotherapy, and the intervention condition in the MI-SPRINT trial, potentially moderated the association of the within-person change in cardiac symptoms with the change in total PTSD symptoms from 3 to 12 months. The rationale for these exploratory analyses was as follows. Patients with greater peritraumatic threat perceptions might perceive cardiac symptoms as more threatening after hospitalization, which could contribute to an increase in PTSD symptoms. In contrast, patients engaging in cardiac rehabilitation or psychotherapy or those receiving trauma-focused counseling might gain more knowledge and confidence in dealing with cardiac symptoms. This would potentially reduce the magnitude and valence of symptom threat, leading to fewer PTSD symptoms.

## Methods

### Study Design and Participants

For this study, we analyzed data of all 156 participants in the Myocardial Infarction-Stress Prevention Intervention (MI-SPRINT) randomized controlled trial who underwent a clinical interview to assess ACS-induced PTSD symptoms 3 and/or 12 months after ACS (8, 9). The MI-SPRINT trial was a single-center study conducted at a university hospital in Switzerland. The trial's primary aim was to test whether one single session intervention of trauma-focused counseling, delivered within 48 h of hospital admission, prevents the development of interviewer-rated ACS-induced PTSD symptoms compared with stress-focused counseling (active control intervention) at 3 months (21). In brief, trauma-focused counseling applied an educational approach, targeting individual patients' resources and cognitive (re)structuring to prevent any MI-induced traumatic reactions that might occur in the weeks to come. Stress-focused counseling comprised information about the general role of psychosocial stress in cardiac disease, but any trauma-related terminology was strictly avoided. Both conditions were single-blinded active face-to-face interventions of equal duration (i.e., 45 min) and attention, delivered at the bedside in coronary care unit rooms (21). Although we could not previously show a benefit for trauma-focused counseling when compared with stress-focused counseling (8, 9), we included the intervention condition as a covariate in the current study.

Consecutive patients were included in the MI-SPRINT trial if they were 18 years or older, had a verified ACS, either acute ST segment elevation MI (STEMI) or non-STEMI, stable circulatory conditions, and a high level of distress during ACS, including peritraumatic threat perceptions. The latter was defined by a score of at least 5 for pain intensity plus at least 5 for fear of dying and/or feelings of helplessness on numeric rating scales ranging from 0 to 10. We excluded patients who received emergency cardiac surgery, those with severe comorbid diseases, limited orientation, cognitive impairment, current severe depression based on the cardiologist's clinical judgement, suicidal ideations in the last 2 weeks, insufficient knowledge of German to comprehend study instructions, and those participating in another trial.

Based on a power analysis detailed elsewhere (21), the MI-SPRINT trial had a target simple size of *n* = 388, which could not be achieved, primarily because of the large number of patients who were discharged before they could be approached by study staff (8). Between 2/2013 and 9/2015, 190 patients were enrolled, of whom 97 (51.1%) were randomized to trauma-focused counseling and 93 (48.9%) to stress-focused counseling. Of the 156 patients who completed the study, 104 provided data at both assessments, 50 at 3 months only, and 2 at 12 months only. The 156 completers of the study were on average 5.2 years younger than the 34 non-completers (*p* = 0.014), but there were no significant group differences in the other baseline characteristics. Attrition was similar in the two intervention groups leaving slightly more patients in the trauma-focused counseling group at 3 months (80 vs. 74) and 12 months (59 vs. 47). Recruitment, reasons for dropouts (the main reason being lack of institutional funding to conduct the 12-month follow-up in 50 participants), and the content of the intervention have been described elsewhere (8, 9). The ethics committee of the State of Bern, Switzerland, approved the study protocol, which was registered under ClinicalTrials.gov (NCT01781247). All participants provided written informed consent.

### Measures

#### Timing of Data Collection

At hospital admission, research assistants obtained measures from patient files and through a structured medical history. At 3 and 12 months, patients were invited for a clinical interview to assess PTSD symptoms and to self-rate depressive symptoms. A structured medical history was performed at 3 and 12 months to assess health behaviors, therapeutic interventions, and medical events that had occurred between hospital discharge and 3 months and between 3 and 12 months after ACS. Cardiac symptoms that had occurred in the previous 4 weeks were also assessed at 3 and 12 months.

#### Symptoms of Post-traumatic Stress Disorder

We used the validated German version of the Clinician-Administered PTSD Scale (CAPS) with reference to Diagnostic and Statistical Manual for Mental Disorders (DSM)-IV criteria (23) to assess ACS-induced PTSD symptoms at 3 and 12 months. Symptom items were anchored to the MI that had led to the index acute hospitalization as the traumatic event. An interviewer rated the frequency and intensity of each of the 17 PTSD symptoms in the prior month, using a 5-point (0–4) Likert-type rating scale. Frequency (0 = “never,” 1 = “once or twice,” 2 = “once or twice a week,” 3 = “several times a week,” 4 = “daily or almost every day”) and intensity (0 = “none,” 1 = “mild,” 2 = “moderate,” 3 = “severe,” 4 = “extreme”) ratings are summed for each symptom to yield a severity score ranging from 0 to 8. Accordingly, the total PTSD symptom severity score ranges from 0 to 136. The re-experiencing cluster includes 5 symptoms, the avoidance/numbing cluster 7 symptoms and the hyperarousal cluster 5 symptoms. Accordingly, severity scores range from 0 to 40 for re-experiencing symptoms and hyperarousal symptoms and from 0 to 56 for avoidance/numbing symptoms. For the CAPS total severity score, scores ≥20 indicate clinically significant PTSD symptoms (24) and a difference of 10 points corresponds to a minimal clinically important difference (25). Cronbach's α for the CAPS total severity score was 0.79 at 3 months and 0.71 at 12 months. Respective values were 0.68 and 0.63 for re-experiencing scores, 0.66 and 0.56 for avoidance/numbing scores and 0.44 and 0.33 for hyperarousal scores.

#### Cardiac Symptoms

As the bulk of research on interoceptive accuracy investigates cardiac signal with tests of heartbeat detection (26), we decided to examine the effects of heartbeat symptoms and chest tightness/pain separately and to combine them only in a second step. A timeframe of 1 month has been recommended for recall of common somatic symptoms with acceptable reliability (27). Therefore, at both assessments, participants were asked if they had experienced chest tightness or chest pain in the last 4 weeks (yes/no) and to indicate if these occurred at rest and/or during physical exertion. As it can be difficult for some patients to distinguish “chest tightness” from “chest pain,” both symptoms were offered together as alternative responses for a category of subjective complaints localized to the chest area. For the assessment of a category of heartbeat symptoms, participants were asked whether they had experienced heart palpitations, racing of the heart, heart stumbling and/or the heart skipping a beat in the last 4 weeks. We coded a “yes” answer if at least one of these symptoms had occurred. Participants were also asked to answer “yes” or “no” to the occurrence of each of the four heartbeat symptoms individually. A measure of total cardiac symptom burden was calculated by summing individual cardiac symptoms present (i.e., chest tightness/pain at rest, chest tightness/pain during exertion and the 4 heartbeat symptoms; score range 0–6). Similar questions about cardiac symptoms have been used in research on enduring somatic threat in patients with ACS (14).

#### Time-Invariant Covariates

Time-invariant variables were assessed at hospital admission. The intervention condition was included as a covariate. Demographic variables included age and sex. MI type (i.e., STEMI or non-STEMI that had led to the index acute hospitalization), and the number of coronary vessels diseased (i.e., luminal narrowing ≥50%), were used as indices of ACS severity. Resuscitation prior to hospital arrival and/or in the hospital were lumped in one category “resuscitation.” The duration between pain/symptom onset and hospital admission was categorized (code) as <24 h (“1”), >24–48 h (“2”), >48–72 h (“3”), >72 h-7 days (“4”) and >7 days (“5”). As described under inclusion criteria, pain intensity during ACS was self-rated with a numeric scale. Peritraumatic threat perceptions was calculated as the mean of numerical ratings, each ranging from 0 to10, for fear of dying and helplessness perceived during ACS. Based on the Charlson comorbidity index, three categories were formed for low, intermediate, or high 10-year mortality risk (28). Probable PTSD cases due to traumatic experiences in the 3 months prior to the index acute hospitalization were explored with a 3-item screener (29). Depression history was assessed asking patients the question “Have you ever had a depression in your life? (yes/no).” The body mass index (BMI) was calculated based on self-reported weight and height (kg/m^2^).

#### Time-Varying Covariates

Time-varying covariates were assessed at 3 and 12 months. Depressive symptoms were self-rated with the 21-item Beck Depression Inventory (BDI) (30). Each item is scored with a value between 0 and 3, referring to the severity of the depressive symptom, resulting in a total score between 0 and 63. Patients were categorized into either current smokers or former/never smokers. Physical activity per week “that makes you sweat” was categorized as none, 1–2x or 3–7x. Based on the J-shaped association between the amount of alcohol consumed and cardiovascular risk, alcohol consumption was categorized as moderate (“0”), none (“1”) or heavy (“2”) (>21 standard drinks/week for men and >14 for women) (31). The number of emergency visits to the hospital, family physician and/or other medical specialists were asked from participants as well as if they were hospitalized due to a cardiac event or a cardiac invasive procedure (e.g., re-stenting, pacemaker implantation). Medical records were consulted to verify the information if available. Participants were also asked whether they had participated in cardiac rehabilitation and/or psychotherapy, and if so, to what extent (i.e., the number of weeks of inpatient or outpatient cardiac rehabilitation and of psychotherapy sessions).

### Statistical Analyses

Data management and analyses were conducted in SPSS 27.0 for Windows (SPSS Inc., Chicago, IL) with a significance level of *p* < 0.05 (2-tailed). Descriptive statistics are reported as means (M) and standard deviations (SD), median and inter-quartile range (IQR) or *n* (%). We used multiple imputation (k = 5) to replace missing values for time-invariant covariates. There were 3 missing values for BMI, 9 for PTSD screen and 3 for depression history. Missing values for time-varying covariates were replaced by the median of the respective assessment, which is an appropriate procedure if missing values are <5% of a sample and the data are skewed. Overall, there were 11 (4.2%) missing values for BDI scores, 1 (0.4%) for weeks of rehabilitation, and 3 (1.2%) for the number of psychotherapy sessions. One patient who had no psychotherapy in the first 3 months after ACS and who missed this information for the 3–12-month interval was classified as “no psychotherapy.” Due to a non-normal distribution, all CAPS scores (dependent variables) were square-root transformed prior to analysis. This power transformation was selected based on a comparison of the shape of the distribution for other transformations.

The Wilcoxon test and McNemar test (as appropriate) were performed to test for a difference in time-varying variables among the 104 participants who contributed data to both the 3- and 12-month assessments. Linear mixed-effects models analysis with random intercepts and restricted maximum likelihood estimation was conducted to examine the longitudinal association of chest tightness/pain and heartbeat symptoms with total PTSD symptoms and PTSD symptom clusters, adjusting for covariates. When both the dependent variable and the predictor are time-varying, the resultant parameter estimates can be interpreted as the within-person change in the dependent variable (i.e., square-root transformed CAPS scores) associated with the change in the predictor (e.g., cardiac symptoms) from 3 to 12 months. Univariable analysis was conducted to examine the individual association between each covariate and total PTSD symptoms. Fixed effects estimates are reported with 95% confidence intervals (CI). Covariates were selected a priori based on potential associations with PTSD symptoms (22) and entered as fixed effects. Intervention condition, age, sex, MI type, number of coronary vessels diseased, duration between pain onset and hospital admission, resuscitation, peritraumatic threat perceptions, pain intensity during ACS, comorbidity index, PTSD screen at admission, history of depression and BMI were entered as time-invariant covariates. Depressive symptoms, smoking status, alcohol consumption, physical activity, cardiac rehabilitation, psychotherapy, hospitalization due to recurrent cardiac events/invasive cardiac procedures, and the number of emergency visits were entered as time-varying covariates. A covariate “Time” was entered to account for a within-person change in PTSD symptoms between the two assessments. “Time” was coded as “0” for 3-month CAPS scores and “1” for 12-month CAPS scores. In the primary analysis, chest tightness/pain (yes/no) and heartbeat symptoms (yes/no) in the last 4 weeks were entered together in the model as time-varying predictor variables to estimate their association with PTSD symptoms independently of each other. Exploratory analyses using interaction terms examined effect modification of the association between changes in cardiac symptoms and changes in total PTSD symptoms from 3 to 12 months by four factors that could moderate (i.e., amplify or attenuate) cardiac symptoms. These factors were peritraumatic threat perceptions, cardiac rehabilitation and psychotherapy, including the “dosage” of the latter two, and intervention condition. Of further interest was whether a change in total cardiac symptom burden was independently associated with a change in total PTSD symptoms from 3 to 12 months.

## Results

### Participant Characteristics

Table 1 shows the characteristics of the study participants at admission, 3 months, and 12 months after ACS. The sample was predominantly male (84.6%) and had an average age of 59 years. There were a total of 260 assessments after 3 months (*n* = 154) and 12 months (*n* = 106). In the subsample of 104 participants who completed both follow-up assessments, more patients participated in cardiac rehabilitation during the first 3 months after ACS (*p* < 0.001). An equal proportion attended psychotherapy during both periods (*p* = 1.00), but the number of psychotherapy sessions was higher in the first 3 months after ACS (*p* = 0.019). Recurrent cardiac events/invasive cardiac procedures requiring hospitalization occurred more often in the first 3 months after ACS (*p* < 0.001). Chest tightness/pain (*p* = 0.25) and heartbeat symptoms (*p* = 0.11) in the previous 4 weeks were reported equally frequently at 3 and 12 months. However, total cardiac symptom burden (i.e., the sum of all 6 cardiac symptoms present) was greater at 3 months than at 12 months (*p* = 0.033). At the individual symptom level, heart palpitations were the only symptom that occurred more frequently in the first 3 months (*p* = 0.017). Accordingly, the proportion of participants reporting no cardiac symptoms at all in the previous 4 weeks was greater at 12 months than at 3 months (*p* = 0.030).

**Table 1:** Characteristics of study participants at hospital admission and at the two assessments.

**Variables**	**Admission (*n* = 156)**	**3-Month follow-up (*n* = 154)**	**12-Month follow-up (*n* = 106)**	***P***
***Time-invariant***				
Trauma-focused counseling, *n* (%)	81 (51.9)			
Age, years, M (SD)	59.0 (11.1)			
Sex, female, *n* (%)	24 (15.4)			
ST-elevation myocardial infarction, *n* (%)	112 (71.8)			
Coronary vessels diseased, M (SD)	1.90 (0.86)			
Pain/symptom onset				
<24 h, *n* (%) >24-48 h, *n* (%) >48-72 h, *n* (%) >72 h-7 days, *n* (%) >7 days	126 (80.8) 11 (7.1) 7 (4.5) 6 (3.8) 6 (3.8)			
Resuscitation, *n* (%)	12 (7.7)			
Peritraumatic threat, M (SD)	5.30 (1.79)			
Pain during ACS, M (SD)	7.85 (1.66)			
Comorbidity index				
High risk, *n* (%) Medium risk, *n* (%) Low risk, *n* (%)	27 (17.3) 41 (26.3) 88 (56.4)			
PTSD screen positive, *n* (%)	15 (9.6)			
Depression history, *n* (%)	45 (28.8)			
Body mass index, kg/m^2^, M, (SD)	27.9 (4.7)			
***Time-varying***				
CAPS total score, median (IQR)		8.0 (3.0, 15.0)	8.0 (3.75, 14.0)	0.153
CAPS re-experiencing, median (IQR)		2.0 (0, 3.25)	0 (0, 3.25)	0.204
CAPS avoidance/numbing, median (IQR)		2.0 (0, 4.0)	2.0 (0, 5.0)	0.148
CAPS hyperarousal, median (IQR)		4.0 (2.0, 7.25)	4.0 (2.0, 7.0)	0.218
Depressive symptoms, score, median (IQR)		4.0 (2.0, 8.0)	4.0 (2.0, 7.0)	0.962
Current smoker, *n* (%)		20 (13.0)	13 (12.3)	0.453
Alcohol consumption				
Moderate, *n* (%) None, *n* (%) Heavy, *n* (%)		108 (70.1) 37 (24.0) 9 (5.9)	78 (73.6) 23 (21.7) 5 (4.7)	0.506
Physical activity				
None, *n* (%) 1–2x/week, *n* (%) 3–7x/week, *n* (%)		38 (24.7) 20 (13.0) 96 (62.3)	29 (27.4) 28 (26.4) 49 (46.2)	0.217
Cardiac rehabilitation, *n* (%)		130 (84.4)	40 (37.7)	<0.001
Cardiac rehabilitation duration in weeks, median (IQR)		8.0 (3.0, 11.0)	0 (0, 3.0)	<0.001
Psychotherapy, *n* (%)		39 (25.3)	25 (23.6)	1.000
Psychotherapy sessions, median (IQR)		0 (0, 1.0)	0 (0, 0)	0.019
Cardiac hospitalization, *n* (%)		52 (33.8)	13 (12.3)	<0.001
Emergency visits, number, median (IQR)		0 (0, 0.25)	0 (0, 1.0)	0.591
Chest tightness/pain, *n* (%)		69 (44.8)	42 (39.6)	0.248
Chest tightness/pain, rest, *n* (%)		50 (32.5)	36 (34.0)	0.742
Chest tightness/pain, exertion, *n* (%)		46 (29.9)	25 (23.6)	0.307
Heartbeat symptoms, *n* (%)		35 (22.7)	19 (17.9)	0.108
Heart palpitations, *n* (%)		25 (16.2)	10 (9.4)	0.017
Racing of the heart, *n* (%)		7 (4.5)	5 (4.7)	1.000
Heart stumbling, *n* (%)		17 (11.0)	11 (10.4)	0.791
Skipping of a heartbeat, *n* (%)		5 (3.2)	3 (2.8)	0.727
Chest tightness/pain and heartbeat symptoms				
None, *n* (%) Either one category, *n* (%) Both categories, *n* (%)		70 (45.4) 64 (41.6) 20 (13.0)	59 (55.7) 33 (31.1) 14 (13.2)	0.030
Number of cardiac symptoms, median (IQR)		1.0 (0, 2.0)	0 (0, 2.0)	0.033

*ACS, acute coronary syndrome; CAPS, Clinician-Administered PTSD Scale; IQR, interquartile range; M, mean; PTSD, Post-traumatic stress disorder; SD, standard deviation. P-values indicate the level of significance for differences in time-varying variables between the 3-month and 12-month assessments in the 104 participants who contributed data to both assessments using Wilcoxon test or McNemar's test, as appropriate*.

### Post-traumatic Stress Symptoms

Based on a CAPS total score ≥20, the prevalence of clinically significant ACS-induced PTSD symptoms was 18.2% (*n* = 28) at 3 months and 8.5% (*n* = 9) at 12 months. However, the median of the CAPS total score was 8 at both assessments (Table 1) with a non-significant “time” effect in linear mixed models (Table 2), indicating that continuous scores for total PTSD symptoms did not change significantly between 3 and 12 months. This was also the case for each of the three PTSD symptom clusters (Tables 1, 3) and in the subsample of the 104 patients with CAPS scores at both 3 months and 12 months (Table 1).

**Table 2:** Relationships with total PTSD symptoms over 12 months.

	**Univariable**		**Multivariable**	
**Parameter**	**Estimate**	**95% CI**	**Estimate**	**95% CI**
Intercept	2.749^***^	2.517, 2.981	0.886	−1.197, 2.969
Time	−0.200	−0.457, 0.057	0.085	−0.229, 0.398
Trauma-focused counseling	0.225	−0.240, 0.690	0.059	−0.265, 0.384
Age	−0.027^*^	−0.048, −0.006	−0.009	−0.025, 0.007
Female sex	0.665^*^	0.031, 1.298	−0.147	−0.602, 0.309
ST-elevation MI	0.147	−0.368, 0.663	0.098	−0.276, 0.472
Coronary vessels diseased	0.240	−0.030, 0.509	0.084	−0.109, 0.276
Pain/symptom onset	−0.041	−0.268, 0.185	0.033	−0.122, 0.187
Resuscitation	0.411	−0.461, 1.284	0.376	−0.217, 0.969
Peritraumatic threat perceptions	0.318^***^	0.197, 0.438	0.163^***^	0.069, 0.275
Pain during ACS	−0.041	−0.181, 0.010	−0.058	−0.155, 0.039
Comorbidity index	−0.032	−0.335, 0.271	0.095	−0.116, 0.305
PTSD screen positive	0.092	−0.707, 0.892	0.147	−0.409, 0.704
Depression history	0.789^**^	0.288, 1.289	0.152	−0.225, 0.530
Depressive symptoms	0.156^***^	0.121, 0.191	0.111^***^	0.078, 0.144
Body mass index	−0.027	−0.076, 0.023	−0.006	−0.041, 0.029
Current smoker	−0.118	−0.708, 0.471	−0.230	−0.691, 0.231
Alcohol consumption	−0.109	−0.421, 0.204	−0.166	−0.421, 0.089
Physical activity	0.112	−0.099, 0.323	0.135	−0.052, 0.322
Cardiac rehabilitation	0.334^*^	0.013, 0.654	0.244	−0.108, 0.596
Psychotherapy	1.413^***^	0.999, 1.826	0.702^***^	0.308, 1.096
Cardiac hospitalization	0.290	−0.074, 0.659	0.030	−0.326, 0.385
Emergency visits	0.399^**^	0.158, 0.639	0.152	−0.066, 0.371
Chest tightness/pain	0.919^***^	0.564, 1.273	0.588^***^	0.275, 0.900
Heartbeat symptoms	1.122^***^	0.715, 1.529	0.548^**^	0.165, 0.931

*ACS, acute coronary syndrome; MI, myocardial infarction, PTSD, Post-traumatic stress disorder. Total PTSD symptoms (dependent variable) were entered as square-root transformed values of the Clinician-Administered PTSD Scale total score. Pain/symptom onset was treated as a continuous variable. Estimates are unstandardized regression coefficients with 95% confidence interval (CI)*.

*Significance level*:

*** *P < 0.001*;

** *P < 0.010*;

* *P < 0.05*.

**Table 3:** Multivariable relationships with PTSD symptom clusters over 12 months.

	**Re-experiencing**		**Avoidance/numbing**		**Hyperarousal**	
**Parameter**	**Estimate**	**95% CI**	**Estimate**	**95% CI**	**Estimate**	**95% CI**
Intercept	−0.149	−2.032, 1.734	0.478	−1.438, 2.394	0.259	−1.404, 1.922
Time	−0.047	−0.331, 0.236	0.022	−0.264, 0.308	0.076	−0.172, 0.323
Trauma-focused counseling	0.032	−0.261, 0.323	0.055	−0.244, 0.354	−0.047	−0.306, 0.213
Age	−0.004	−0.019, 0.010	0.003	−0.012, 0.018	−0.009	−0.022, 0.004
Female sex	−0.058	−0.467, 0.354	−0.149	−0.569, 0.271	−0.068	−0.433, 0.297
ST-elevation MI	0.185	−0.152, 0.523	0.001	−0.344, 0.345	0.086	−0.213, 0.386
Coronary vessels diseased	−0.010	−0.184, 0.163	0.047	−0.129, 0.224	0.101	−0.052, 0.255
Pain/symptom onset	−0.002	−0.141, 0.138	0.029	−0.114, 0.172	0.033	−0.091, 0.157
Resuscitation	0.271	−0.265, 0.806	0.338	−0.209, 0.884	0.102	−0.373, 0.577
Peritraumatic threat perceptions	0.106^*^	0.021, 0.191	0.071	−0.016, 0.158	0.079^*^	0.003, 0.154
Pain during ACS	−0.014	−0.101, 0.074	−0.077	−0.166, 0.013	−0.004	−0.082, 0.073
Comorbidity index	−0.008	−0.199, 0.182	0.171	−0.024, 0.365	0.041	−0.128, 0.210
PTSD screen positive	0.210	−0.293, 0.713	0.281	−0.232, 0.794	−0.064	−0.509, 0.381
Depression history	−0.029	−0.370, 0.312	−0.128	−0.476, 0.220	0.356^*^	0.054, 0.658
Depressive symptoms	0.057^***^	0.027, 0.087	0.068^***^	0.038, 0.099	0.071^***^	0.044, 0.097
Body mass index	<0.001	−0.031, 0.032	−0.007	−0.039, 0.025	−0.001	−0.028, 0.027
Current smoker	−0.059	−0.476, 0.358	0.032	−0.391, 0.456	−0.252	−0.620, 0.116
Alcohol consumption	0.056	−0.174, 0.287	−0.317^**^	−0.552, −0.083	−0.058	−0.262, 0.145
Physical activity	0.155	−0.014, 0.324	−0.183^*^	−0.359, −0.012	0.307^***^	0.158, 0.456
Cardiac rehabilitation	0.090	−0.228, 0.409	0.135	−0.187, 0.457	0.134	−0.145, 0.413
Psychotherapy	0.330	−0.027, 0.686	0.458^*^	0.096, 0.820	0.422^**^	0.108, 0.735
Cardiac hospitalization	0.006	−0.315, 0.328	−0.090	−0.416, 0.235	0.128	−0.154, 0.410
Emergency visits	0.065	−0.132, 0.263	0.175	−0.025, 0.375	0.034	−0.140, 0.207
Chest tightness/pain	0.450^**^	0.167, 0.733	0.287^*^	0.001, 0.574	0.244	−0.004, 0.493
Heartbeat symptoms	0.392^*^	0.045, 0.739	0.513^**^	0.161, 0.864	0.355^*^	0.051, 0.659

*ACS, acute coronary syndrome; MI, myocardial infarction, PTSD, Post-traumatic stress disorder. PTSD symptoms (dependent variable) were entered as square-root transformed values of the Clinician-Administered PTSD Scale symptom cluster scores. Pain/symptom onset was treated as a continuous variable. Estimates are unstandardized regression coefficients with 95% confidence interval (CI)*.

*Significance level*:

*** *P < 0.001*;

** *P < 0.010*;

* *P < 0.05*.

### Associations With Changes in PTSD Symptoms From 3 to 12 Months

#### Total PTSD Symptoms

Table 2 shows that several variables were significantly associated with changes in total PTSD symptoms from 3 to 12 months in the univariable analysis only. More specifically, younger age, female sex, and depression history were associated with a higher level of PTSD symptoms (at both 3 and 12 months). Moreover, an increase in the number of emergency visits from 3 to 12 months was associated with an increase in total PTSD symptoms, whereas patients who participated in cardiac rehabilitation at 3 months, but not at 12 months, showed a decrease in total PTSD symptoms. In the multivariable model, there were independent associations [estimate (95% CI)] of peritraumatic threat perceptions with a higher level of PTSD symptoms (at both 3 and 12 months) [0.163 (0.069, 0.275), *p* < 0.001] and of changes in depressive symptoms [0.111 (0.078, 0.144), *p* < 0.001], psychotherapy [0.702 (0.308, 1.096), *p* < 0.001], chest tightness/pain [0.588 (0.275, 0.900), *p* < 0.001] and heartbeat symptoms [0.548 (0.165, 0.931), *p* = 0.005] from 3 to 12 months with changes in total PTSD symptoms. Separate analyses predicting the untransformed CAPS score showed a 3.02-point (1.11, 4.92) and 4.35-point (2.03, 6.67) increase in the CAPS total score in patients who reported chest/tightness pain and heart rhythm problems at 12 months but not at 3 months.

#### PTSD Symptom Clusters

Table 3 shows the multivariable models for the three PTSD symptom clusters. With respect to changes in heartbeat symptoms, independent associations emerged with changes in re-experiencing [0.392 (0.045, 0.739), *p* = 0.002], avoidance/numbing [0.513 (0.161, 0.864), *p* = 0.004] and hyperarousal [0.355 (0.051, 0.659), *p* = 0.022]. Change in chest tightness/pain from 3 to 12 months was independently associated with changes in re-experiencing [0.450 (0.167, 0.733), *p* = 0.027] and avoidance/numbing [0.287 (0.001, 0.574), *p* = 0.049], but not hyperarousal. Furthermore, peritraumatic threat perceptions were significantly and independently associated with a higher level (both a 3 and 12 months) of re-experiencing [0.106 (0.021, 0.191), *p* = 0.015] and hyperarousal [0.079 (0.003, 0.154), *p* = 0.041], but not of avoidance/numbing. Changes in depressive symptoms, health behaviors, and psychotherapy from 3 to 12 months showed varying degrees of significant independent associations with changes in PTSD symptom clusters.

### Additional Exploratory Analyses for Changes in Total PTSD Symptoms

First, we explored whether variables that could amplify or attenuate cardiac symptoms would moderate the association of changes in chest tightness/pain or heartbeat symptoms from 3 to 12 months with changes in total PTSD symptoms by adding interaction terms to the multivariable model. There was a significant interaction between psychotherapy and heartbeat symptoms [−0.813 (−1.553, −0.074), *p* = 0.031], but not between psychotherapy and chest tightness/pain (*p* = 0.75). In patients who attended psychotherapy, the association between the onset of heart beat symptoms from 3 to 12 months and an increase in total PTSD symptoms was weaker [0.437 (−0.178, 1.052), *p* = 0.16] than in those who did not attend psychotherapy [0.825 (0.341, 1.309), *p* < 0.001]. The “dosage” of psychotherapy did not account for this relation, as the interaction maintained significance with further adjustment for the number of psychotherapy sessions attended [−0.868 (−1.610, −0.126), *p* = 0.022]. No significant interactions emerged between changes in chest tightness/pain or heartbeat symptoms and peritraumatic threat perceptions (*p* > 0.11), cardiac rehabilitation (*p* > 0.85), and intervention condition (*p* > 0.51).

Second, we explored whether changes in total cardiac symptom burden from 3 to 12 months would show an independent association with changes in total PTSD symptoms. For this purpose, instead of chest tightness/pain (yes/no) and heartbeat symptoms (yes/no), we included the cardiac symptom score in the multivariable model, which showed a significant association [0.343 (0.202, 0.484), *p* < 0.001]. Separate analyses predicting the untransformed CAPS score showed a 2.32-point (1.47, 3.17) increase in the CAPS total score with each additional cardiac symptom that occurred from 3 to 12 months. Thus, a participant who reported no cardiac symptoms at 3 months, but had all 6 symptoms at 12 months, would be predicted to exhibit a 13.91-point (8.79, 19.03) increase in their CAPS total score between 3 and 12 months.

## Discussion

### Main Findings for PTSD Symptoms

We found that a change in the presence or absence of cardiac symptoms between 3 and 12 months after ACS was independently associated with a change in total ACS-induced PTSD symptoms. We accounted for a range of covariates, which have been associated with ACS-induced PTSD symptoms in previous research (22), suggesting that this longitudinal association is robust. Specifically, a probable PTSD diagnosis at admission, a previous depressive episode and a concurrent change in depressive symptoms did not account for the association. Furthermore, the effect size of the positive association between changes in cardiac and PTSD symptoms from 3 to 12 months indicates potential clinical significance. For example, participants who reported neither chest tightness/pain nor heartbeat symptoms at 3 months, but reported both at 12 months, would be predicted to exhibit a 7.4-point increase in their CAPS total score. This magnitude approaches the minimal clinically important difference of 10 points (25). Similarly, a patient who reported all 6 cardiac symptoms at 3 months, but none at 12 months, would be predicted to exhibit a 13.9-point decrease in their CAPS total score when all other covariates in the model remained constant.

Changes in cardiac symptoms from 3 to 12 months were also independently associated with changes in the three PTSD symptom clusters, except for a non-significant association between chest tightness/pain and hyperarousal. If the onset of cardiac symptoms in the post-hospital period contributes to an increase in PTSD symptoms, such an association may have implications for adverse outcomes in patients with ACS-induced PTSD (32). Re-experiencing symptoms may particularly worsen prognosis in patients after ACS, likely through an association with cardiovascular risk factors and atherothrombotic processes (4). Possible mechanisms involved include endothelial dysfunction and hypercoagulability (33) as well as increased inflammation (34). Regarding the latter, re-experiencing symptoms are associated with hyperactivation of the amygdala (35), and enhanced amygdala activation in response to threatening stimuli has been related to increased systemic levels of inflammation in humans (36).

We acknowledge that based on the design of our study, we are unable to make causal inferences about the direction of the association between changes in cardiac symptoms and changes in PTSD symptoms in patients after ACS. However, there are longitudinal studies suggesting that this relation is largely bi-directional (11, 37). Alterations in interoceptive signaling may be one mechanism by which an increase in PTSD symptoms may have contributed to increased perception of cardiac symptoms. Activation of physiological stress axes in traumatized individuals may lead to inaccurate perception of interoceptive body signals (38). In PTSD, cardiac interoceptive accuracy, reflecting correspondence between the perceived and the actual heartbeat signal, is reduced (39). An interoceptive deficit may cause patients with traumatic experience of ACS to perceive their heartbeat and other interoceptive signals like pain sensations as threatening. For instance, interoceptive inaccuracy might result in misinterpretation of physical responses during exercise, such as accelerated heartbeat, as hyperarousal symptoms. To support this, we found that an increase in physical activity from 3 to 12 months was associated with an increase in hyperarousal symptoms. In turn, to reduce the triggered threat perception, patients could avoid physical activity (6), which may explain why we found that a decrease in physical activity from 3 to 12 months was associated with an increase in avoidance/numbing symptoms in our sample.

### Potential Implications for Future Intervention Studies

Before our main findings can be translated into clinical practice, the components of successful interventions for cardiac symptoms need further research, for which we offer some considerations based on previous studies and those of our exploratory analyses. We found that participants who attended psychotherapy at 12 months, but not a 3 months, exhibited an increase in PTSD symptoms, which could be an expression of greater illness burden. Alternatively, this suggests that patients may experience greater PTSD symptom reduction if they start psychotherapy early rather than later after ACS. Moreover, it is a promising finding that in patients with onset of heartbeat symptoms from 3 to 12 months, those who attended psychotherapy showed a weaker increase in total PTSD symptoms than those not attending psychotherapy. We can only speculate about potential mechanisms or if the use of psychotherapy is simply a marker for better outcome in those with onset of heartbeat symptoms. The number of psychotherapy sessions did not explain this effect, and whether a possible influence of cardiac symptoms on PTSD symptoms was specifically addressed in psychotherapy is unknown. However, short-term cognitive behavioral therapy, including exposure to physical activity, has been effective for the treatment of non-cardiac chest pain and palpitations in outpatients (40). Therefore, similar interventions could be tailored to patients with cardiac symptoms in an attempt to reduce ACS-induced PTSD symptoms.

Based on our study findings, and research showing that greater physical symptom burden predicted more severe PTSD symptoms in military personnel deployed to combat (37), the mere reduction of the number of cardiac symptoms in the post-hospital period could be effective in reducing ACS-induced PTSD symptoms. An obvious intervention would be rigorous anti-ischemic treatment in accordance with guidelines to reduce chest tightness/pain during exertion (41), reported by one in four participants in our sample.

Heartbeat detection tasks have paved the ground for research on (cardiac) interoceptive accuracy (42) and, as discussed above, there is a bi-directional longitudinal association between cardiac symptoms and PTSD symptoms. Therefore, interoceptive training for more accurate sensing of signals concerning the internal state of the body, particularly the heartbeat, could decrease cardiac symptoms in the post-hospital phase in patients with ACS-induced PTSD symptoms.

In contrast, we found no evidence that patients with cardiac symptoms might benefit from trauma-related counseling in terms of PTSD symptom level. Possible explanations are that a 45-min session did not provide enough time for an effective trauma-related intervention or that comprehensive information on stress management made the control condition equally effective in managing cardiac symptoms.

### Strengths and Limitations

The repeat assessment of clinician-rated PTSD symptoms, cardiac symptoms and other time-varying covariates, such as health behaviors and therapeutic interventions, which may change in the post-hospital phase, is a strength of our study. However, in addition to the considerable drop-out rate in terms of available 12-month follow-up data due to lack of funding, there are other notable limitations. The findings of our study may not be generalizable to patients with low levels of peritraumatic threat perceptions, as fear of dying or helplessness of at least medium intensity were inclusion criteria for the study. Although peritraumatic threat perceptions were directly associated with the level of PTSD symptoms (at both 3 and 12 months), its reduced variance may partially explain why it did not moderate the association of changes in cardiac symptoms with changes in PTSD symptoms. We did not ask participants how scared or worried they were about their cardiac symptoms. Consideration of cardiac threat perception could have even increased the strength of the association between changes in cardiac symptoms with changes in PTSD symptoms. We were unable to address the role of cardiac symptoms that might have arisen in the first 2 months post-hospitalization. The results for hyperarousal symptoms must be interpreted with caution because the internal consistency of the hyperarousal score was problematically low. Good internal consistency is commonly reported in studies of different types of trauma (24), although to our knowledge there are no other studies that have used the CAPS to assess hyperarousal symptoms in patients after ACS, which precludes comparison with the results of our study. The number of covariates controlled in the analysis was large for a relatively modest sample size, but univariable analysis confirmed that changes in cardiac symptoms from 3 to 12 months were associated with changes in PTSD symptoms without adjustment for covariates. Exploratory analyses without adjustment for multiple testing carry the risk of incidental findings, but may also generate valuable hypotheses that can be tested in future studies. We analyzed data from a predominantly male sample with relatively high educational attainment, low comorbidity, and no major current depressive episode, originally recruited for an intervention study. However, the prevalence of clinically significant ACS-induced PTSD symptoms was similar to previous observational studies (2). Finally, as we applied DSM-IV criteria for PTSD that were in effect at the time when we planned the study, it is unclear whether the results would hold with DSM-5 criteria.

## Conclusion

The findings of our study suggest that in patients with ACS, a change in chest tightness/pain and heartbeat symptoms from 3 to 12 months after ACS is independently associated with a change in ACS-induced PTSD symptoms. The results are consistent with the growing literature that physical symptoms are an important clinical entity to consider in comprehensive care for traumatized individuals as they may predict PTSD symptoms. It seems worthwhile to test in longitudinal studies whether interventions that improve interoceptive accuracy related to threat perception of cardiac symptoms can reduce the development of ACS-induced PTSD symptoms.

## Data Availability Statement

The anonymized raw data supporting the conclusions of this article will be made available by the authors to qualified researchers, without undue reservation.

## Ethics Statement

The studies involving human participants were reviewed and approved by Ethics Committee of the State of Bern, Switzerland. The patients/participants provided their written informed consent to participate in this study.

## Author Contributions

RvK, US, HZ, JB, and J-PS conceived and designed the study. RvK performed statistical analysis. RvK, MP, and RM-L handled funding and supervision. RM-L and MP acquired the data. RvK, JB, AP, and US drafted the manuscript. HZ, CZ-H, J-PS, MP, and RM-L made critical revision of the manuscript for key intellectual content. All authors approved the final version of the manuscript for submission.

## Funding

This study was financially supported by grant No. 140960 from the Swiss National Science Foundation. Additional support came from the Teaching and Research Directorate, Bern University Hospital, Switzerland.

## Conflict of Interest

The authors declare that the research was conducted in the absence of any commercial or financial relationships that could be construed as a potential conflict of interest.

## Publisher's Note

All claims expressed in this article are solely those of the authors and do not necessarily represent those of their affiliated organizations, or those of the publisher, the editors and the reviewers. Any product that may be evaluated in this article, or claim that may be made by its manufacturer, is not guaranteed or endorsed by the publisher.
